# Actæa Racemosa

**Published:** 1873-11

**Authors:** F. H. Bailey

**Affiliations:** Knoxville, Tenn.


### ACTÆA RACEMOSA.

BY F. H. BAILEY, M. D., OF KNOXVILLE, TENN.

This is a plant well known in the United States, and variously called Cimicifuga, Racemosa, and Black Snakeroot. Its use as a medicine appears to be principally confined to the United States and the Canadas. I have been familiar with it from my first commencement of study. Dr. Horace Green used it freely in his early practice, and in his "*Selections of Prescriptions,*" it is mentioned in several formulæ. It does not appear to have been known to English writers except from American sources of information. Pereira mentions it in the American edition of his Materia Medica, (1846). Neligan, in his edition of 1844, alludes to it merely from American authority. It is in rheumatism that it has been mostly used in this country, and I have for that affection, as well as in chronic ailments of the hepatic function, used it with favorable results.

I generally combine it with iod. potassii, and other agents which may be indicated at the same time. It can be used in acute rheumatism, combined with alkaline salts, ipecac and sanguinaria canadensis. In sub-acute and chronic rheumatism I have made free use of it in connection with tonics and tonic alteratives.

In chronic affections of the liver, where there is a dull, aching pain complained of in the right and upper part of that organ, attended with the usual pain at the top of the shoulder, I have given actæa freely, and am in almost the daily habit of prescribing it in this locality, where such an affection is very common.

Dr. Green alludes to its use in chorea, and it is doubtless in cases where that disease is a concomitant or sequel of rheumatism, that it has been successful.

The following is a favorite formula with me in any non-inflammatory condition of the liver:

℞. Tr. actæa racemosæ, ℥ ij.; Iod. potassii, ℥ ij.; Sys. ipecac ℥ j.; Tr. sang. canad., ℥ ss. M. Sig. Teaspoonful three times daily.

In acute rheumatism I use a similar mixture, giving less of the actæa and more of the ipecac.

Very often I combine tr. cinchona, or sulph. quinine, according to conditions and indications.

In reviewing the literature upon this medicine, I find that in the December number of the Chicago *Medical Examiner*, there is an article of my own, in which the use of actæa was beneficial in a case of rheumatism followed with decided symptoms of chorea.

The patient, a young lady of seventeen, was attacked with rheumatism of the right foot and ankle. By the employment of iod. potassii, pulv. doveri, and spts. nit. dulc., the extremity became better, but chorea soon made its appearance in the same limb. On the second day the muscles on the right side of the face were involved, and it appeared eminent that chorea might become fully developed. I accordingly prescribed as follows:

℞. Fluid ext. valerian, Fluid ext. act. racemosæ, aa ℥ j.; Syr. iod. ferri, ℥ ss.; Sul. quinine, ℈j. To be taken in doses of a teaspoonful three times daily.

The convulsive movements soon began to disappear, but continued to be apparent after she was able to walk about.

After about two months, during which time she continued to take the mixture, all traces of the affection disappeared. If I recollect rightly, however, the young lady became affected with cardiac symptoms, suffering from palpitation and becoming pale and anæmic in appearance.

---

Twenty dollars a day is the amount of fine incurred by the law of Nova Scotia, going in force on the first of May, for practicing medicine without being registered.

